# The Experience sampling method in psychiatry: a state-of-the-art lecture

**DOI:** 10.1192/j.eurpsy.2024.30

**Published:** 2024-08-27

**Authors:** I. Myin Germeys

**Affiliations:** Dept of Neurosciences, KU Leuven, Leuven, Belgium

## Abstract

**Abstract:**

Psychiatric problems occur in people’s normal daily life, in a dynamic interaction with the context people are in. Yet, we have very few techniques to assess this dynamical nature of symptoms, nor do we have good insights in how people actually function in their ordinary life. Ambulatory assessment techniques such as Experience Sampling Methodology (ESM) or Ecological Momentary Assessment (EMA) have been proposed as a potential clinical tool to bridge this gap. Yet, very few of these techniques have actually made it to the clinic. In my talk, I will discuss the strengths and limitations of using these digital diary techniques to open up someone’s real life in clinical practice. I will discuss qualitative research identifying barriers and facilitators, identified by patients and clinicians. I also will discuss what is needed in terms of technology and data security, by demonstrating the MoMent app and MoMent Dashboard, that has been developed within the H2020 IMMERSE project. Finally, I will discuss how these digital mental health tools could help in developing a much more fine-grained understanding of how psychopathology emerges in the realm of ordinary life, making patients active partners in the clinical process.

**Disclosure of Interest:**

None Declared

